# Similarities in the Metabolic Reprogramming of Immune System and Endothelium

**DOI:** 10.3389/fimmu.2017.00837

**Published:** 2017-07-21

**Authors:** Chu-Yik Tang, Claudio Mauro

**Affiliations:** ^1^Barts and The London School of Medicine and Dentistry, Institute of Health Sciences Education, Queen Mary University of London, London, United Kingdom; ^2^Barts and The London School of Medicine and Dentistry, William Harvey Research Institute, Queen Mary University of London, London, United Kingdom

**Keywords:** metabolism, metabolic reprogramming, glycolysis, oxidative phosphorylation, macrophage, T lymphocyte, endothelial cell

## Abstract

Cellular metabolism has been known for its role in bioenergetics. In recent years, much light has been shed on the reprogrammable cellular metabolism underlying many vital cellular processes, such as cell activation, proliferation, and differentiation. Metabolic reprogramming in immune and endothelial cells (ECs) is being studied extensively. These cell compartments are implicated in inflammation and pathogenesis of many diseases but their similarities in metabolic reprogramming have not been analyzed in detail. One of the most notable metabolic reprogramming is the Warburg-like effect, famously described as one of the hallmarks of cancer cells. Immune cells and ECs can display this phenotype that is characterized by a metabolic switch favoring glycolysis over oxidative phosphorylation (OXPHOS) in aerobic conditions. Though energy-inefficient, aerobic glycolysis confers many benefits to the respiring cells ranging from higher rate of adenosine triphosphate production to maintaining redox homeostasis. Chemical and biological regulators either promote or perturb this effect. In this review, nitric oxide, hypoxia-inducible factor, and adenosine monophosphate-activated protein kinase have been discussed for their common involvement in metabolic reprogramming of both systems. From *in vitro* and animal studies, various discrepancies exist regarding the effects of those regulators on metabolic switch. However, it is generally accepted that glycolysis favors inflammatory reactions while OXPHOS favors anti-inflammatory processes. The reasons for such observation are currently subject of intense studies and not completely understood. Finally, metabolic reprogramming in immune cells and ECs does not limit to the physiological state in health but can also be observed in pathological states, such as atherosclerosis and cancer. These new insights provide us with a better understanding of the similarities in metabolic reprogramming across a number of cell types, which could pave the way for future research and possible metabolic-based therapeutics.

## Introduction

Cellular metabolism has been sidelined for many years and it was only seen as the series of sequential pathways involved in converting fuel sources, such as glucose, fatty acids, ketones, and amino acids to generate packets of cellular energy in the form of adenosine triphosphate (ATP). At its infancy, scientists found it difficult to convince the scientific community that cellular metabolism and its by-products might have wider implications on inflammation and disease pathogenesis (1). In 1997, Shim et al. conducted a study using representational difference analysis and found that lactate dehydrogenase (LDH)-A induction by the oncogene c-myc leads to overproduction of lactate (2). This finding provides a molecular explanation to the metabolism of tumor cells characterized by glucose overutilization and lactate overproduction in normal oxygen conditions, also known as the Warburg effect. This reprogramming of cellular energetics in cancer cells is described as one of the ten hallmarks of cancer by Douglas Hanahan and Robert A. Weinberg in 2011 (3). With regard to lactate, it was recently found that the high levels of lactate present in the inflammatory microenvironment reduce the motility of T cells, hence serving an active retention mechanism and lead to the induction of T helper (T_H_)17 responses, which suggests that lactate is not merely a by-product of cellular metabolism, but it has a role in determining specific immune cell responses (4). There is an increasing awareness that many fundamental cellular processes, such as cell differentiation and proliferation have their distinct but reprogrammable metabolic requirements, which many experts would coin as the “renaissance of cellular metabolism.”

At the heart of these exciting findings, there is a growing interest in explaining well-established disease pathogenesis in the light of metabolic reprogramming. Inflammation is a tissue response to insult by host defense mechanisms. Although the inflammation is the bodily response to rid invading pathogens and promote healing of injured tissues, often unresolved chronic inflammation or dysregulated autoimmunity can be the core of many disease processes. In inflammation, endothelial cells (ECs) are responsible for controlling immune cell trafficking. Cells of the immune system are recruited to the site of injury *via* dilated tissue vasculature. With the lower shear stress in dilated vessels, immune cells accumulate at the margins of the lumen in proximity to the endothelium. The immune cells roll along and adhere firmly on the endothelium before migrating across the vessel in a process known as diapedesis. There is a major interplay between immune cells and ECs to orchestrate these complex series of events. Extensive studies on the metabolic reprogramming in both cells types are underway but little or no comparisons between these closely related systems exist in the current literature. Hence, this review will analyze and compare the similarities in metabolic reprogramming of endothelial and immune cells in both health and disease state and further discuss about possible therapeutic targets.

## Metabolic Characteristics of Macrophages, T Cells, and ECs

### Macrophage Metabolism

Macrophages have been traditionally described by two differentiation pathways, which lead to the classical (M1) and the alternative (M2) phenotype (5). M1 and M2 phenotypes, however, are now known to belong to a spectrum of possible differentiation pathways alongside with numerous activation states with characteristic phenotypes. Recently, plaque-specific macrophages, such as M4, Mhem, and Mox, have been identified in atherosclerosis lesions (6–9). M2 macrophages can further be classified into M2a, M2b, M2c, and M2d subtypes (6, 10, 11). Xue et al. stimulated human macrophages with 28 different activation stimuli and analysis of the data has shown a spectrum of macrophage activation states which does not conform to the classical bipolar M1/M2 axis (12). Despite the existence of a myriad of macrophage subsets, the M1/M2 paradigm is still extensively used in the literature and is a useful framework for the purpose of our discussion pertaining to macrophage metabolism and function.

In general, M1 macrophages are pro-inflammatory and they are functionally important for clearance of pathogens. M1 macrophage metabolism is characterized by high glycolysis and relatively low oxidative phosphorylation (OXPHOS), high inducible nitric oxide synthase (iNOS) activity, and nitric oxide (NO) production (13). The tricarboxylic acid (TCA) cycle of M1 macrophages is discontinuous and it has two break-points at isocitrate dehydrogenase and succinate dehydrogenase (SDH) (14). This observation provides explanation for the upstream accumulation of citrate and succinate, both of which influence the polarity of macrophages. Succinate acts as a pro-inflammatory signaling molecule by stabilizing hypoxia-inducible factor (HIF), a transcription factor which upregulates the biosynthetic capacity of cells (15). Citrate is also a known pro-inflammatory signal. Citrate is involved in fatty acid and phospholipid biosynthesis that promotes the production of inflammatory prostaglandins (16). In the cytosol, the metabolism of citrate by ATP-citrate lyase generates nicotinamide adenine dinucleotide phosphate (NADPH) which is a required substrate for the synthesis of NO (17). Unlike naïve cell types, macrophages are terminally differentiated and they do not require energy for proliferation (18). Instead ATPs are used to sustain energy-demanding cellular activities, such as phagocytosis and secretory functions (18).

M2 macrophages, on the other hand, are involved in regenerative roles, such as tissue remodeling, repair, and healing. M2 macrophage metabolism is characterized by OXPHOS, fatty acid oxidation (FAO), and upregulated arginase 1 activity (13). Unlike M1 macrophages, the mitochondrial complexes in M2 macrophages are not occupied by NO and reactive oxygen species (ROS) and, hence, OXPHOS is sufficient to sustain the metabolic demand. M2 macrophages also express PFKFB1, an isoform of 6-phosphofructo-2-kinase capable of metabolizing the glycolytic activator fructose-2,6-bisphosphate (19). As opposed to the upregulated pentose phosphate pathway (PPP) in M1 macrophage, PPP is suppressed in M2 macrophage by the expression of carbohydrate kinase-like protein (20). l-arginine metabolism *via* arginase produces ornithine, which is important for the synthesis of proline, a component of tissue collagen (21). Consequently, arginase activity might be driving the reparative function of M2 macrophages in tissue remodeling. Lastly, the predilection for OXPHOS and FAO in M2 macrophage is also driven by high adenosine monophosphate-activated protein kinase (AMPK) activity (22).

### T Cell Metabolism

The role of T cells in the adaptive immune system is vast and they function by secreting lymphokines to induce immunomodulatory actions (CD4^+^) or by promoting cytotoxicity (CD8^+^). CD4^+^ T cells bind to major histocompatibility complex (MHC) class II ligands on antigen-presenting cells, while the counterpart, CD8^+^ T cells, bind to MHC class I ligands. There are several subtypes of CD4^+^ T cell differentiation, including T_H_1, T_H_2, T_H_17, T_H_9, regulatory T (T_reg_) cell, follicular helper T cell, type 1 T regulatory cell, and memory T cells (23–25).

Naïve T cells rely on FAO and OXPHOS for their energy production (26, 27). Upon encountering antigen-presenting cells, naïve T cells undergo activation and subsequently clonal expansion and differentiation. During this period, profound metabolic changes occur. Activated T cells upregulate aerobic glycolysis and the glycolytic branch reaction, PPP (28, 29). This is attained *via* glucose transporter (GLUT) 1 translocation to cell periphery and upregulation of glycolytic enzymes (28, 30). Glutamine metabolism is also enhanced which supplies products for the TCA cycle as well as promoting polyamine synthesis (29, 31).

Distinct metabolic pathways are required for differentiation of activated T cells. The T effector subsets, T_H_1, T_H_2, and T_H_17, are known to prefer glycolysis even in aerobic conditions (32, 33). T_reg_ cells, on the other hand, mainly prefer FAO for energy generation and replicate at moderate levels as opposed to the profound amplification in cell proliferation seen in T effector cells (18, 33, 34). Metabolism of memory T cells is largely similar to that in naïve T cells, aside from the increased mitochondrial mass that is a preemptive measure to prepare for mitochondrial energy generation upon secondary antigen exposure (35).

### EC Metabolism

Endothelial cells can be classified into three different subtypes based on their morphology and role in angiogenesis: the highly branched tip cells are primarily migratory and navigate the direction of the vessel sprout; the stalk cells have less branches but are highly proliferative and elongate the sprout during extension; and lastly, phalanx cells, commonly recognized by their cobblestone appearance, are quiescent and line the mature blood vessels (36). In maturity, majority of the blood vessels are relatively quiescent but they retain the capacity to respond to angiopoietin in physiological and pathological states to generate new blood vessels from existing vasculature, in a process known as angiogenesis (37). This sequential and highly coordinated process can be accomplished by either sprouting or non-sprouting angiogenesis (37, 38).

**Figure 1:**
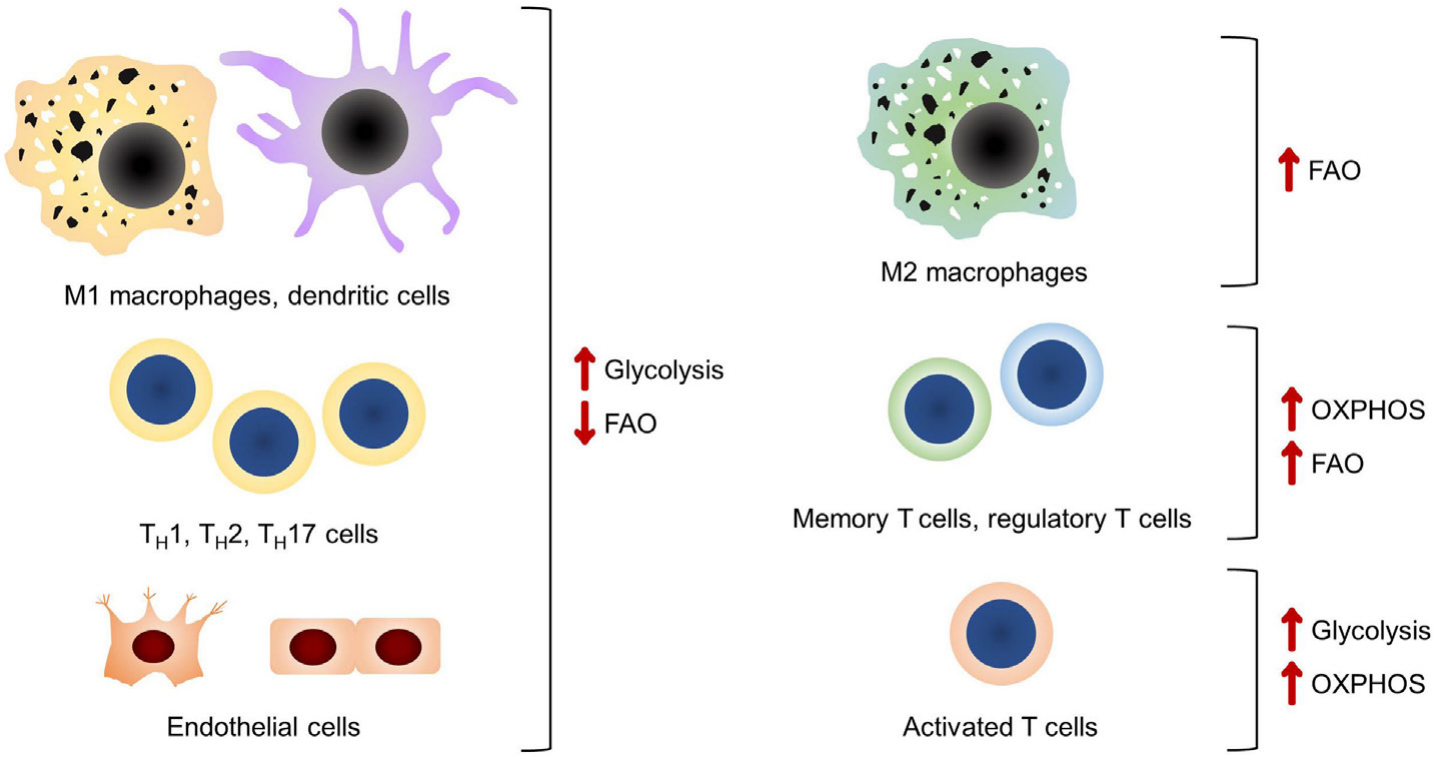
A summary of the main bioenergetic pathways in each of the cell types discussed. It should be noted that glycolysis is the preferred bioenergetic pathway in classical pro-inflammatory cells and endothelial cells.

Owing to the low mitochondrial content, ECs rely almost exclusively on glycolysis for energy generation (39). On vascular endothelial growth factor (VEGF) stimulation, the rate of glycolysis is doubled and GLUT 1 expression is upregulated to meet the metabolic demand of cell migration during angiogenesis (40, 41). PFKFB3, a different isoform to PFKFB1 expressed in M2 macrophages, is also involved in the metabolic regulation of ECs. PFKFB3 stimulates glycolysis by synthesizing large quantity of fructose-2,6-bisphosphate, which is a potent glycolytic stimulator (39). Glycolysis is shown to modulate the phenotypic expression of ECs. Increased activity of PFKFB3 could override genetically predestined stalk cells into metabolically active tip cells to further enhance ECs sprouting (39). Conversely, extracellular environment such as laminar shear stress exerted by blood flow lowers PFKFB3 activities and the associated metabolic changes that resultantly sustain cellular quiescence (42). Apart from angiogenesis, ECs are involved in a wide range of vascular homeostatic functions such as vasodilation and the anti-proliferating effect on vascular smooth muscle cells through the action of NO generated by endothelial nitric oxide synthase (eNOS) (43). As in the case with M2 macrophages, AMPK in ECs senses glucose deprivation and promotes the inhibition of acetyl-coenzyme A carboxylase (ACC), resulting in increased FAO (44). Schoors et al. investigated FAO in ECs and found it essential for ECs proliferation during vessel sprouting, unlike glycolysis that regulates both proliferation and migration (45). The targeted control of FAO on ECs proliferation could potentially be a therapeutic option for pathological angiogenesis (Figure 1).

## Metabolic Reprogramming in the Immune Cells and ECs

Cells derive energy from catabolism of the three major biomass sources: glucose, fatty acids, and amino acids mainly through glycolysis and OXPHOS. In theory, a molecule of glucose can yield up to approximately 38 molecules of ATP of which, 2 from glycolysis, 2 from the TCA cycle, and 34 from OXPHOS. Based on the number of moles of ATP produced per mole of glucose, OXPHOS would be the ideal and most efficient bioenergetic pathway. However, glycolysis is the preferred one in cells with high metabolic demands as it confers many benefits. The demand for an expensive amount of energy from metabolically active processes can be met by aerobic glycolysis, which is a faster bioenergetic pathway as compared to OXPHOS, granted that there is a steady stream of glucose supply (46). The metabolic intermediates of glycolysis can also be shunted to branch reactions and synthetic pathways to generate macromolecules and maintain redox homeostasis needed for cell proliferation, migration, and maintaining physiological functions (17, 41, 47). M1-activated macrophages rely on glycolysis for ATP production, while the mitochondrial machinery has been shunted to ROS production necessary for bactericidal activity (48). The enhanced glycolytic branch reaction PPP also augments the production of NADPH required for the production of ROS (49). In CD4^+^ T cells, it was shown that OXPHOS and aerobic glycolysis can interchangeably be used to sustain cell proliferation and survival, but only aerobic glycolysis can adequately lead to the attainment of full effector function (26, 31). ECs invariably utilize glycolysis as the main bioenergetic process and several advantages of aerobic glycolysis have been suggested. Despite the proximity to readily available oxygen sources, glycolysis reduces the need for oxygen and this allows maximum oxygen concentration carried in the blood to tissues perfused by the blood vessels (18). In neovascularization, ECs proliferate in order to perfuse hypoxic tissues. Low oxygen level in hypoxic environment limits the effectiveness of OXPHOS in generating ATP and, hence, glycolysis is preferred (18). Reaction–diffusion models show that the decrease in partial pressure of oxygen is less than the decrease in glucose concentration over distance (18, 50). This suggests that ECs can still undergo anaerobic glycolysis as tip cells get further away from blood vessels. In this review, NO, HIF, and AMPK will be discussed in more detail owing to their common involvement in the regulation of the Warburg-like effect and other forms of bioenergetics seen in all three cell types. Other potent metabolic regulators, such as mTOR, are not within the scope of this review as there is limited literature of its effect on ECs (18, 41). The main similarities and differences in the metabolic pathways of macrophages, T cells, and ECs are summarized in Table 1.

**Table 1:** The similarities and differences in metabolic reprogramming mediated by nitric oxide (NO), hypoxia-inducible factor (HIF), and adenosine monophosphate-activated protein kinase (AMPK) in macrophages, T lymphocytes and endothelial cells (ECs).

	Macrophages	T lymphocytes	ECs
NO	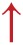 Glycolysis, 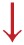 oxidative phosphorylation (OXPHOS) (17, 51, 52)	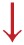 Glycolysis (53) 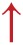 OXPHOS (53, 54) 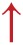 Effector T cell apoptosis (55) 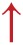 Regulatory T (T_reg_) population (56)	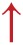 Glycolysis, 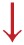 OXPHOS (57, 58) 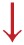 Glyceraldehyde-3-phosphate dehydrogenase activity (59)
HIF	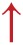 Glycolysis (60–63)	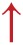 Glycolysis (29, 64) 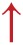 T_h_17 differentiation, 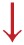 T_reg_ population (65)	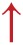 Glycolysis (66, 67)
AMPK	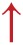 Fatty acid oxidation (FAO), 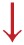 inflammatory response (68, 69) 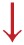 M1 macrophage polarization (70) 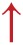 Glycolysis in monocytes (71)	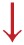 Glycolysis (72) 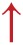 OXPHOS (73)	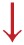 Glycolysis (74) 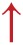 FAO (74)

*On a whole, the metabolic changes induced by these metabolic signals are similar between the immune and endothelial systems. However, NO suppresses glycolysis in T cells, while AMPK upregulates glycolysis in monocytes, both of which do not conform to the general framework*.

### Nitric Oxide

Synergistic stimulation of macrophages by lipopolysaccharide (LPS) and interferon (IFN) has been shown to increase the expression of iNOS and NO production and resulting in a preferential switch to glycolysis (17, 51, 52). One of the possible mechanisms is *via* the nitrosylating effect of NO on iron-sulfur proteins of the electron transport chain complex leading to inhibition of cell respiration (75, 76). iNOS-deficient dendritic cells are unresponsive to LPS stimulation but pharmacological reintroduction of exogenous NO results in a metabolic switch from OXPHOS to aerobic glycolysis (75). Several studies have shown that NO can permeate cell membranes to exert its effect on cellular metabolism. Recent studies have shown that low plasma membrane cholesterol and functional connexin-based channels facilitate the transport of NO across cellular membranes (77, 78). These observations suggest that NO generated by ECs could exert its effect on immune cells in a paracrine fashion. In terms of cellular production of NO, the substrate l-arginine can be shunted into two metabolic processes, either the iNOS or the arginase pathway. iNOS metabolizes l-arginine to produce NO and citrulline while arginase, on the other hand, controls the ornithine cycle by catalyzing the formation of ornithine and urea. Both enzymes compete for the same common substrate and, hence, NO production and its effect on metabolism are controlled by the expression of these enzymes. Colegio et al. found that lactate generated from aerobic glycolysis could induce the expression of arginase 1 and M2-like phenotype in tumor-associated macrophages (TAMs) *via* the HIF pathway (79). This finding has implications for cancer cell survival and is further discussed in a later section.

A study on murine thymus shows that the nitrosylating effect of NO is also seen in other enzymes regulating the activity of glycolysis, the TCA cycle, and fatty acid metabolism (80). NO has a toxic effect on T cells, consistent with a role as self-limiting pathway in T_H_1 cell-mediated responses *in vivo via* IFN-γ (56, 81). In an experimental study on autoimmune disorder, IFN-γ exerted a protective effect *via* activation of iNOS and production of NO (82). NO in turn triggered T cell apoptosis, thus providing some degree of explanation to the self-limiting effector response of T_H_1 cells (55). Addition of l-arginine to CD4^+^ T cells was shown to induce several metabolic changes, including increased gluconeogenesis, decreased GLUTs and glycolytic enzymes, and a metabolic switch to OXPHOS (53). A possible explanation is the upregulation of the serine biosynthetic pathway which fuels the TCA cycle and subsequently OXPHOS and results in a “reverse” Warburg-like effect (53, 54). T cells have also been shown to display a twofold decrease in glycolytic rate when iNOS is knocked-out (83). Alongside the apparent anti-inflammatory effect of NO on T cells, it was shown that NO induces proliferation of functional T_reg_ cell population (56). The metabolic mechanism behind the increased proliferation of T_reg_ cells by NO is not well understood but it may represent a key regulation point of host immune function.

Nitric oxide is arguably the most important synthetic product of ECs as it has a role in modulating vasodilation and inflammation. eNOS is functionally similar to iNOS and it metabolizes l-arginine to citrulline and NO. Aside from its local effect on vascular smooth muscle cells, NO is also an autocrine signaling molecule and exhibits effects on ECs metabolism. In the angiogenic state, the metabolic demand of ECs is greatly increased and this is met by increasing the rate of glycolysis (39–41). Using an NO-donor, it was shown that glyceraldehyde-3-phosphate dehydrogenase (GAPDH), a glycolytic enzyme in bovine aortic ECs is inhibited in a dose-dependent manner but the effect is reversed to near base-line upon removal of the NO-donor, which implies that GAPDH is a target for NO-mediated oxidative stress (59). The same group of researchers conducted another study and found that NO stimulates glycolysis and reversibly impairs mitochondrial reserve capacity (57). This set of data opposes the earlier finding on NO-mediated inhibition of GAPDH, leading to the conclusion that inhibition of this enzyme is not a sensitive biological effect of NO (57). Other studies have also been in support of the action of NO in promoting glycolysis in ECs. Paik et al. treated human umbilical vein ECs with sodium nitroprusside and diethylenetriamine, both of which are NO donors and found that ^18^F-fluorodeoxyglucose uptake was increased alongside with GLUT 1 expression and hexokinase activity (58). These changes are surrogate markers of increased glycolytic flux in ECs. Despite not many studies support a role for NO-mediated effects *via* rewiring of EC metabolism, NO produced from eNOS is theoretically capable of interacting with the immune system and, hence, it represents a topic of interest for further investigation.

### Hypoxia-Inducible Factor

*In vitro* and *in vivo* studies show that glycolytic flux is upregulated by both M1 activation and hypoxic condition (60). Cellular adaptation to hypoxia is mediated by the transcription factor HIF (84). It is made of two subunits, HIF-1β and HIF-1α of which the latter is degraded by oxygen-dependent mechanisms (84). HIF-1α expression is induced by T_H_1 cytokine stimulation and is known to promote the metabolic switch from OXPHOS to glycolysis by regulating the activity of GLUT 1 and several enzymes in the glycolytic pathway (61). HIF-1α also increases LDH and reduces pyruvate dehydrogenase activity, collectively shunting the production of acetyl-CoA for the TCA cycle to the production of lactate inducing the Warburg-like effect in macrophages (62, 63). Interestingly, Takeda et al. found that in T_H_1 cytokine-induced M1 macrophages, HIF-1α induces the expression of iNOS but not arginase, while the converse is true for HIF-2α in M2 macrophages (85).

Wang et al. found that glycolysis, glutamine metabolism, and FAO were not affected by short-term (24 h) deletion of HIF-1α but mild glycolysis impairment was shown after 72 h with moderate downregulation of LDH-A and hexokinase-2 gene expression (29). Despite the data suggesting a limited role of HIF-1α in promoting the reprogramming of T cell metabolism, HIF-1α-deficient CD4^+^ T cells cultured in T_H_17-stimulating conditions show lower expression of genes encoding for GLUTs, LDH-A, and other glycolytic enzymes (64). Interestingly, HIF has also been shown to regulate the T_H_17/T_reg_ balance by favoring T_H_17 differentiation and suppressing T_reg_ population (65). This is also apparent in T_H_17-mediated autoimmune disease as *in vivo* studies showed that mice with HIF-1α-knock out T cells were resistant to experimental autoimmune encephalitis (65). It is known that T_H_17 cells favor glycolysis, while T_reg_ cells rely on OXPHOS and FAO for energy generation. This experiment provides indirect evidence for a possible link between HIF and the Warburg-like effect seen in T cells.

Similar metabolic adaptation to hypoxia is also seen in ECs. An experiment on bovine aorta and human umbilical ECs shows that, in hypoxic conditions, ECs increase glucose uptake and lactate generation, both of which are surrogates for glycolysis activity (66). The team then inhibited OXPHOS under aerobic conditions and an upregulation of GLUTs in ECs was observed over several hours. The authors suggest that the lag period is observed as a result of a series of events involving mRNA transcription and protein translation of the GLUTs (66). Oswald et al. studied the effect of hypoxia on the metabolism of ECs from three different sources, namely umbilical, dermal, and aortic. The expression of HIF and VEGF mRNA as markers of experimental hypoxia was measured and they found that hypoxia is associated with increased uptake of ^18^F-fluorodeoxyglucose by ECs from all tissue sources (67). The authors concluded that the low oxygen tension stabilizes HIF-1α that is involved in the upregulation of GLUT-1 expression (67).

### Adenosine Monophosphate-Activated Protein Kinase

Adenosine monophosphate-activated protein kinase is known as the metabolite-sensing kinase and is activated in conditions of low energy and oxygen reserve (17, 86). The breakdown product of ATP, adenosine monophosphate (AMP), and adenosine diphosphate (ADP) bind to AMPK making it more susceptible to activation by upstream kinases (17, 87). AMPK has been shown to play a role in regulating mitochondrial bioenergetic reactions. One proposed pathway is by directly upregulating mitochondrial biogenesis through the induction of its transcriptional coactivator, proliferator-activated receptor-γ-coactivator-1 that induces mitochondrial biogenesis and respiration (88, 89). AMPK also induces the activity of SDH in the TCA cycle that fuels OXPHOS (90). Another notable function of AMPK is the modulation of mTOR as one of its downstream targets. mTOR acts as an intracellular sensor to metabolic cues and directs the rate of cell growth and proliferation (91). Depletion of ATP activates AMPK that in turns inhibits mTOR and subsequent protein synthesis in order to conserve energy (92). Aside from that, AMPK also has a role in upregulating FAO by inhibiting ACC, a rate-limiting enzyme for the carboxylation reaction of acetyl-CoA to malonyl-CoA (93). However, the study outcomes on the importance of AMPK in FAO have not been always congruent. Recent evidence shows that skeletal muscles with AMPK kinase-dead mutant display ACC phosphorylation, reduction in malonyl-CoA, and FAO rate similar to those in controls (94). Nonetheless, these data are not retrieved from studies on immune cells or ECs but rather conducted in skeletal muscles for their abundance in mitochondria content.

Stimulation by M2 stimuli, such as IL-10 and TGF-β rapidly activates AMPK (70). Inhibition of AMPK by genetic deletion of its subunits leads to a heightened inflammatory response and reduction in FAO, a key bioenergetic pathway in M2 macrophages (68, 69). These observations are in concordance with the role of AMPK in opposing the polarization of M1-activated macrophages (70). In one occasion, AMPK has been shown to increase glycolysis in monocytes during hypoxia by activating the glycolytic stimulator, PFKFB3 (71). O’Neill and Hardie proposed that this observation could be explained as an ATP-generating function of AMPK by activated macrophages during hypoxia (22). Another interesting theory from Luo et al. based on their work on tumor cells describes that AMPK induces glycolysis and FAO in acute stress, whereas chronic AMPK stimulation dampens glycolysis *via* inhibition of mTOR and its action on p53 tumor suppressor protein, albeit the evidence for the latter is limited (95). The relevance of this dual effect of AMPK on the metabolism of macrophages requires further investigation.

Although T_H_1, T_H_2, and T_H_17 subtypes of activated CD4^+^ T cells adopt glycolysis as their main form of energy generation, T_reg_ cells, on the other hand, rely mainly on fatty acid metabolism (18, 33, 34). Metformin, a known inducer of AMPK, was shown to increase the number of T_reg_ cells in murine studies (33). To find out the direct role of AMPK on T_reg_ cells metabolism, genetic methods were employed and it was shown that knock out of the AMPKα1 subunit increases GLUT-1 expression and hexokinase activity (72). This resulted is a threefold increase in basal glycolytic rate observed in T-cells supporting the role of AMPK in hindering the switch to aerobic glycolysis (72). However, the cells did not display any polarization toward pro-inflammatory subtypes despite an increase in glycolysis (72). Mayer et al. found that T cells in AMPK-deficient mice displayed increased rate of cell death and reduced aerobic glycolysis as compared to wild-type T cells when mitochondrial respiration is artificially inhibited, which suggests the role of AMPK as a response mechanism to metabolic stress (96). T cell activation and cytokine production, on the other hand, remain intact in AMPK-deficient mice (96). Blagih et al. conducted another study by subjecting T cells to glucose-scarce environment, which inhibited glycolysis. They found that AMPK is essential for glutamine-dependent OXPHOS when glucose supply is limited, providing further evidence for the role of AMPK in T cell response during metabolic stress (73). Functionally, although AMPK-deficient T cells display full proliferative capacity when glucose supply is not a limiting factor, the same scenario is not true when glucose supply is low (73). *In vivo* experiments show that the T cell population was relatively smaller in AMPK-deficient mice as compared to wild-type controls (73). There was no difference observed in viral load but there was a reduction in bacterial clearance in the AMPK-deficient group (73). Taken together, these observations show that despite the AMPK-induced metabolic reprogramming seen in T cells, AMPK might not display an overt *in vitro* effect on T cell function, such as activation and differentiation. However, AMPK could be essential for T cell function in metabolic stress and *in vivo* environment where the cellular micromilieu is more variable.

**Figure 2:**
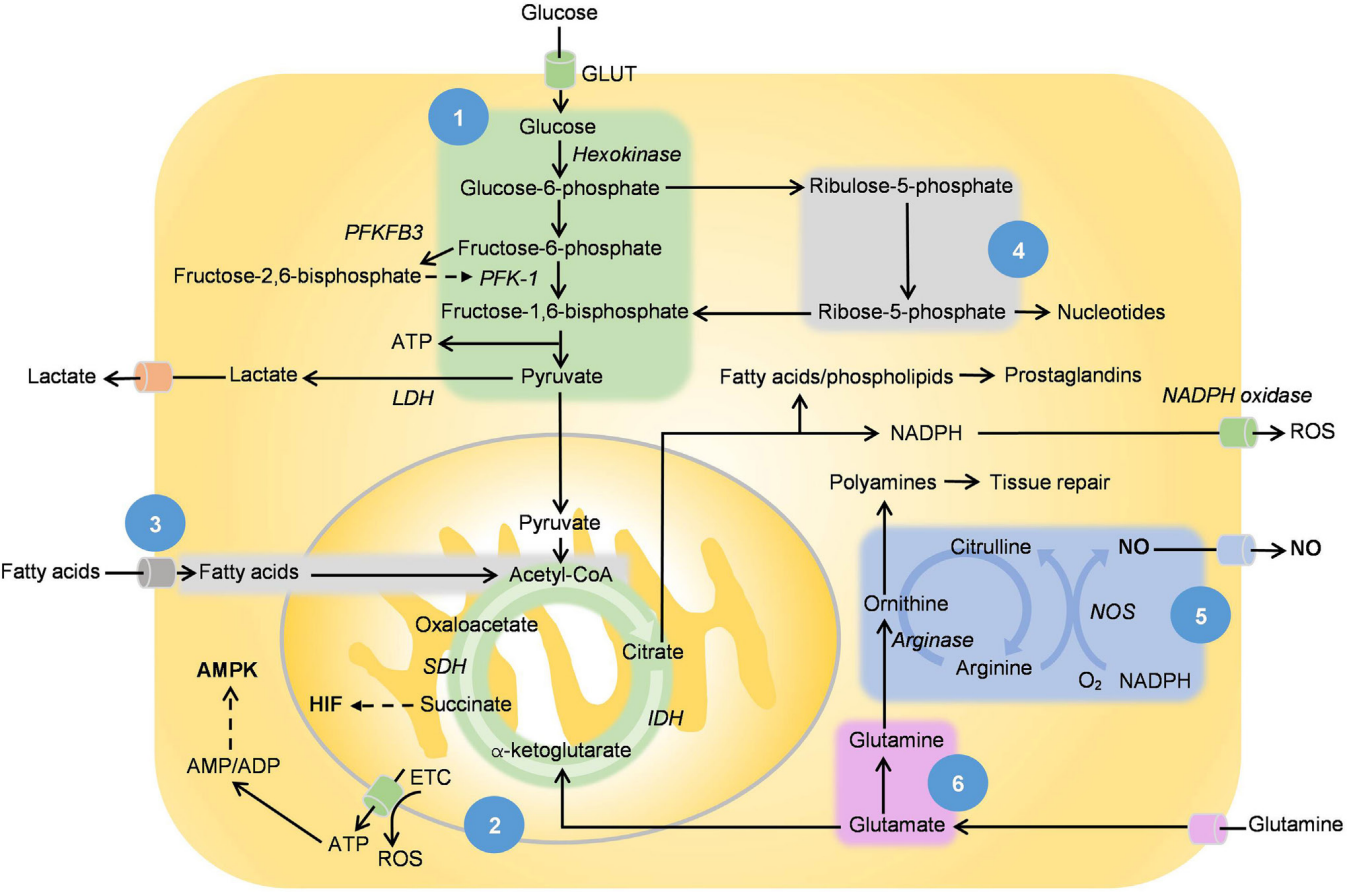
[1] Glycolysis, [2] tricarboxylic acid (TCA) cycle and oxidative phosphorylation, [3] fatty acid oxidation, [4] pentose phosphate pathway, [5] ornithine cycle, and [6] glutamine metabolism. This figure shows the main metabolic pathways in relation with nitric oxide (NO), hypoxia-inducible factor (HIF), and adenosine monophosphate-activated protein kinase (AMPK). NO is produced by NOS with l-arginine as the substrate while succinate, the intermediate of the TCA cycle stabilizes and activates HIF. Adenosine triphosphates (ATPs) are hydrolyzed to adenosine diphosphate (ADP), while some are converted to adenosine monophosphate (AMP) *via* adenylyl cyclase. The increase in AMP/ADP:ATP ratio as well as other extracellular metabolic stressors activate AMPK. The effect of these mediators on metabolic reprogramming is listed in Table 1. PFKFB3 converts fructose-6-phosphate to fructose-2,6-bisphosphate, which in turn activates phosphofructokinase-1 and promotes the rate of glycolysis. Citrate metabolism produces acetyl-CoA, which is converted to malonyl-CoAs for fatty acid synthesis. Arachidonic acid and its derived inflammatory prostaglandins are produced from the same pathway. Arginase regulates the ornithine cycle, which is involved in the production of polyamines, a prominent feature of metabolism in M2 macrophages.

Although the name suggests otherwise, AMP has not been shown to be responsible for the activation of AMPK in ECs. Alternative stimuli, such as the increase in ADP/ATP ratio, tumor suppressor gene product LKB1, shear stress exposure, and Ca^2+^-elevating agonists, such as bradykinin and thrombin, have been proposed (87, 97–99). Similar to the observations seen in M1 macrophages and T_reg_ cells, AMPK activation was shown to reduce glucose uptake and glycolysis in ECs (74). ECs are known to generate up to 85% of their total ATP through glycolysis (39). Therefore, endothelial mitochondria have been proposed to serve other functions other than solely being the energy powerhouse of the ECs. The expression of mitochondrial antioxidant enzymes induced by AMPK has shown to confer protective benefit to ECs against oxidative stress (100). Apart from its effect on glucose metabolism, AMPK activation has been shown to increase FAO in human umbilical vein ECs (74). Specifically, palmitate oxidation is heightened from the activation of AMPK by bradykinin, suggesting that AMPK activation may mitigate lipotoxicity secondary to fatty acid accumulation in the initial stages of atherosclerosis (98) (Figure 2).

## Cellular Metabolism in Inflammation

### Atherosclerosis from a Perspective of Cellular Metabolism

Despite being largely seen as a lifestyle disease, a large part of atherosclerosis stems from inflammation. The earliest evidence of atherosclerosis in a human’s lifetime is fatty streaks, which are inflammatory lesions found in blood vessels, even in those of infants and young children. As described earlier, NO has several homeostatic functions in the circulatory system, including modulating vasodilation, inhibiting platelet aggregation, and smooth muscle proliferation, all of which if dysfunctional will result in atherosclerosis. In ECs, an imbalance of antiatherogenic NO versus its counterpart, the proatherogenic ROS such as superoxides, is often described in early atherosclerosis (101–103). eNOS uncoupling also occurs when l-arginine supply is limited, generating ROS instead of NO (104). On the other hand, administration of l-arginine over 13 weeks has also been shown to regress atherosclerotic lesions in rabbits with hypercholesterolemia, suggesting reversibility in atherosclerosis progression through the restoration of NO action (105). In a study, plasma level of l-arginine, asymmetric dimethylarginine (ADMA), and symmetrical dimethylarginine in 49 patients with hypercholesterolemia were compared with the controls and it was found that increased ADMA associates with impaired vasodilation and reduction in urinary nitrate (106). These effects were shown to be reversible upon l-arginine administration in a double-blinded, randomized, placebo-controlled cross over study and the researchers concluded that ADMA can serve as a novel risk factor for cardiovascular disease (106). It is postulated that ADMA is a competitive inhibitor of NOS, which at high levels results in reduction of NO synthesis (107). This competitive inhibition could then be overcome by increasing the plasma level of l-arginine.

The inflammatory process of atherosclerosis is dependent on the balance of pro-inflammatory and anti-inflammatory cells (108, 109). In early atherogenesis, T_H_1 cytokines, such as IFN-γ, and lipoprotein, activate M1 macrophages that liberate more pro-inflammatory cytokines (110). M2 macrophages, on the other hand, were shown to counteract inflammation and promote healing (21). Regulation of this balance could be a potential therapeutic option for atherosclerosis. Some regulators such as peroxisome proliferator-activated receptor gamma coactivators 1β were shown to induce OXPHOS and FAO in macrophage population and to enhance the maturation of anti-inflammatory M2 macrophages (110). Induction of M2 macrophages can also be achieved *via* AMPK activation (111). Accumulation of M2 macrophages *in vivo* was found to decreases the size of atherosclerotic plaques in mice with hypercholesterolemia (111).

Although most T effector responses aggravate atherosclerosis, T_reg_ cells activity was shown to reduce the size of lesions and extent of inflammation (112, 113). In a murine study, injection of anti-CD3 antibody was shown to induce the T_reg_ cell population and promote regression of atherosclerotic lesions. This beneficial effect was abolished soon after injection of anti-CD25 antibody that depleted T_reg_ cells (114). Macrophages with increased expression of enzymes involved in the synthesis of retinoic acid, such as aldehyde dehydrogenase 1A2 and retinal dehydrogenase were shown to increase T_reg_ cell population (111). This is in tandem with the role of retinoic acid in supporting differentiation of T_reg_ cells from naïve T cells (115).

Patients with cardiovascular risks are classically treated with statins, a drug that inhibits HMG-CoA reductase, a rate-controlling enzyme of the mevalonate pathway that leads to the production of cholesterol. HMG-CoA reductase inhibition *via* statins, however, causes reduced immunosuppressive activity by T_reg_ cells, an effect that could limit the therapeutic effect of statins (116, 117). Restoration of T_reg_ cell function can be achieved through administration of mevalonate, the product of 3-hydroxy-3-methylglutaryl-CoA reductase enzymatic reaction (116, 117). The role of mevalonate as an adjunctive treatment for lipid-lowering statin regimen is also an interesting topic for clinical research. These discoveries are exciting as they open new ways of treating this extensively studied condition, which has plagued the humanity since the beginning of modernization.

### The Survival Mechanisms of Cancer Cells

In neoplasm, growing tumors proliferate at an exceedingly fast rate, rendering the micromilieu deprived of glucose and amino acids essential for T cell function (118–120). This is in concordance with the importance of metabolic pathways in controlling cellular functions. T cells assume an effector status by upregulating glycolytic and anabolic activities through increased glucose uptake, glutamine oxidation, and oxygen consumption (27). The glucose-scarce micromilieu perturbs these energy-expensive processes and sends CD4^+^ T cells into dormancy known as the anergy state and, subsequently, T cell dysfunction (119–121). CD4^+^ T cells that have undergone metabolic inhibition and anergy fail to proliferate even upon re-challenging with costimulation, which is the signal required for T cells to achieve full effector status and maximal expression of cytokines (121). Furthermore, lactate accumulation secondary to tumor glycolysis from the Warburg effect leads to acidosis in the microenvironment (122). High levels of lactate have been shown to inhibit T cell motility and glycolysis *via* distinct transporters exhibited by CD4^+^ and CD8^+^ T cells (4). This inhibition coupled with HIF-induced upregulation of programmed cell death-ligand 1 grants tumor cells immunity against cytotoxic effect of T cells (119, 120, 123). In terms of NO, T cell cytotoxicity can be hindered as a result of peroxynitrite formation from the rapid interaction of ROS and NO generated by tumor iNOS from l-arginine metabolism (118).

To contribute to the existing complexity, M2 TAMs are also implicated in tumor cell survival. As mentioned before, M2 TAMs have heightened arginase activity that converts l-arginine to ornithine and urea, further depleting the limited local source of l-arginine. Ornithine induces the polarization of more M2 TAMs and, hence, this leads to the development of a vicious circle (124). The competition for l-arginine hampers the ability of M1 TAMs to generate NO that has antitumor properties (124). The scarcity of l-arginine also has a knock-on effect on T cell function as they also depend on l-arginine for NO and protein synthesis, both of which are required for T cell activation. In short, tumor cells have developed a distinctive reprogrammed metabolism that provides survival advantage by fast-tracking energy production and anabolic processes while in the process also creates the perfect microenvironment to hinder the metabolism and antitumor function of immune cells.

Tumor ECs, on the other hand, have overexpression of GLUTs, which is indicative of rapid glucose uptake and glycolytic activity (40, 41). Perivascular NO gradient has been shown *in vitro* to facilitate blood vessel normalization and maturation (125). As seen in the competition with immune cells for metabolic substrate, increased uptake of l-arginine by cancer cells reduces NO generated by eNOS. This results in the formation of abnormal vessels in terms of organization, structure, and function, proving a challenge for delivering antitumor drugs to the perfused cancer cells (125). Recently, upregulation of PFKFB3, a key regulator of ECs glycolysis, was shown to lead to the development of immature and dysfunctional vasculature in tumor angiogenesis (39, 41, 126). Further to that, inhibition of the enzyme results in normalization of blood vessels and, hence, it could be a potential therapeutic target as an adjunctive treatment for effective delivery of modern cytotoxic agents to tumor cells (39, 41, 126). At the same time, heightened glycolysis in tumor cells is PFKFB3-dependent and inhibition of the same enzymes was shown to reduce glucose uptake and proliferation of human hematopoietic and adenomatous cancer cell lines (127).

The clinical implication of cell metabolism in oncology is vast. Since the year 2000, lactate accumulations have been reported by several teams as prognostic predictors of poor outcome in patients with solid malignant tumors (128). Several glycolytic inhibitors have also been shown to be effective therapeutic adjuncts against cancers in hypoxic environment and those with mitochondrial defects that are resistant to conventional chemoradiotherapies (129). Although the theoretical advantage of glycolysis inhibitors is to devoid tumor cells of ATP by inhibiting their main mode of bioenergetics, it is postulated that cancer cells with intact mitochondria could still generate ATP through OXPHOS. The other advantage of inhibiting glycolysis is to normalize the acidic tumor micromilieu and the subsequent reestablishment of tumor-suppressing immune function. Arginine metabolism has also attained great interest in cancer therapeutics and, interestingly, both upregulation and inhibition of arginase action can lead to tumor-suppressing activities. Tumor growth displayed dose-dependent suppression on administration of arginase inhibitor and the effect is not seen in mice with dysfunctional adaptive immune system, suggesting that the antitumor activity of arginase inhibitor is immune mediated (130). On the other hand, supplementation of recombinant human arginase was shown to induce cell apoptosis in non-small cell lung cancers through mitochondrial-derived ROS production (131). However, arginase supplementation reduces l-arginine required for the T-cell proliferation and cell–cycle progression from the accumulation of myeloid-derived suppressor cells (MDSC), suggesting a need for co-targeting MDSC accumulation in arginase inhibitor cancer treatments (132). At the moment, the knowledge of cellular metabolism is pushing the boundaries of modern oncology but the *in vivo* effect of metabolic reprogramming remains ambiguous in certain conditions.

## Perspective and Conclusion

The impact of metabolism on cell function is an area with great future prospect for research. For example, NO has been shown to selectively induce differentiation of naïve CD^+^ 4 T cells into T_H_1 phenotype (56). Although both T_H_1 and T_H_2 cells utilize aerobic glycolysis as their main source of bioenergetics, the reasons for NO to selectively induce T_H_1 differentiation are not completely understood (33). It is exciting and equally challenging to find out methods to translate our knowledge on metabolic reprogramming into therapeutics of human diseases. Seeing the fact that bioenergetic mechanisms are ubiquitous in all cell lines, often the big question in therapeutics may be the systemic side effects of metabolic-based treatments as a result of unintended involvement of bystander cells. To illustrate, a partial and reversible reduction of glycolysis can be achieved through PFKFB3 blockade with an intention to reduce proliferation, migration, and sprouting of ECs (133). Potentially, the enzyme blockade could sufficiently reduce pathological progression of atherosclerosis while sparing healthy vasculature from permanent glycolytic dysfunction. Furthermore, pathogenesis of some well-described diseases could be seen from a different light with new understanding in metabolic reprogramming. Recently, novel research on endothelial dysfunction in rheumatoid arthritis is underway (134). ROS is found to be directly implicated in synovitis associated with rheumatoid arthritis (135). Patients with rheumatoid arthritis also have raised plasma ADMA, a known cause of NO-mediated endothelial dysfunction (136). These new findings could lead to the development of treatment adjuncts to couple with the current regimen largely based on immunosuppressants such as methotrexate. These examples are milestones we have achieved in a relatively short span of time, a testimony of the tremendous potential in the field of cellular metabolism. Cellular functions are highly dependent on the metabolic requirement; understanding the metabolic pathways will inform us with novel approaches to exploit these functions in the light of therapeutic and translational opportunities.

## Author Contributions

C-YT and CM conceived and wrote the manuscript.

## Conflict of Interest Statement

The authors declare that the research was conducted in the absence of any commercial or financial relationships that could be construed as a potential conflict of interest.

## Abbreviations

ACC, acetyl-carboxylase A carboxylase; ADMA, asymmetric dimethylarginine; ADP, adenosine diphosphate; AMP, adenosine monophosphate; AMPK, AMP-activated protein kinase; ATP, adenosine triphosphate; CARKL, carbohydrate kinase-like protein; cGMP, cyclic guanosine monophosphate; ECs, endothelial cells; eNOS, endothelial nitric oxide synthase; ETC, electron transport chain; FAO, fatty acid oxidation; GAPDH, glyceraldehyde-3-phosphate dehydrogenase; GLUT, glucose transporter; HIF, hypoxia-inducible factor; HMGCR, 3-hydroxy-3-methylglutaryl-CoA reductase; IDH, isocitrate dehydrogenase; IFN, interferon; iNOS, inducible nitric oxide synthase; LDH, lactate dehydrogenase; LPS, lipopolysaccharide; MHC, major histocompatibility complex; NADPH, nicotinamide adenine dinucleotide phosphate; NO, nitric oxide; OXPHOS, oxidative phosphorylation; PFK, phosphofructokinase-1; PPP, pentose phosphate pathway; ROS, reactive oxygen species; SDH, succinate dehydrogenase; SDMA, symmetrical dimethylarginine; TAMs, tumor-associated macrophages; TCA, tricarboxylic acid; T_H_, T helper; T_reg_, regulatory T; VEGF, vascular endothelial growth factor.
